# Self-managed medication abortion outcomes: results from a prospective pilot study

**DOI:** 10.1186/s12978-020-01016-4

**Published:** 2020-10-27

**Authors:** Heidi Moseson, Ruvani Jayaweera, Sarah Raifman, Brianna Keefe-Oates, Sofia Filippa, Relebohile Motana, Ijeoma Egwuatu, Belen Grosso, Ika Kristianingrum, Sybil Nmezi, Ruth Zurbriggen, Caitlin Gerdts

**Affiliations:** 1 Ibis Reproductive Health, 1736 Franklin Street, Suite 600, Oakland, CA 94612 USA; 2 Department of Epidemiology and Biostatistics, University of California, San Francisco, USA; 3 Ibis Reproductive Health, Cambridge, MA USA; 4 Ibis Reproductive Health, Johannesburg, South Africa; 5 Generation Initiative for Women and Youth, Lagos, Nigeria; 6 La Revuelta Colectiva Feminista, Neuquén, Argentina; 7 Samsara, Yogyakarta, Indonesia

**Keywords:** Abortion, Accompaniment, Africa, Mifepristone, Misoprostol, Self-managed abortion, South America, Southeast Asia

## Abstract

**Background:**

To evaluate the feasibility of conducting a prospective study to measure self-managed medication abortion outcomes, and to collect preliminary data on safety and effectiveness of self-managed medication abortion, we recruited callers to accompaniment groups (volunteer networks that provide counselling through the out-of-clinic medication abortion process by trained counselors over the phone or in-person).

**Methods:**

In 2019, we enrolled callers to three abortion accompaniment groups in three countries into a prospective study on the safety and effectiveness of self-managed medication abortion with accompaniment support. Participants completed up to five interview-administered questionnaires from baseline through 6-weeks after taking the pills. Primary outcomes included: (1) the number of participants enrolled in a 30-day period, (2) the proportion that had a complete abortion; and (3) the proportion who experienced any warning signs of potential or actual complications.

**Results:**

Over the 30-day recruitment period, we enrolled 227 participants (95% of those invited), and retained 204 participants (90%) for at least one study follow-up visit. At the 1-week follow-up, two participants (1%) reported a miscarriage prior to taking the pills, and 202 participants (89% of those enrolled and 99% of those who participated in the 1-week survey) had obtained and taken the medications. Three weeks after taking the medications, 192 (95%) participants reported feeling that their abortion was complete. Three (1.5%) received a surgical intervention, two (1%) received antibiotics, and five (3%) received other medications. Participants did not report any major adverse events.

**Conclusion:**

These results establish the feasibility of conducting prospective studies of self-managed medication abortion in legally restrictive settings. Further, the high effectiveness of self-managed medication abortion with accompaniment support reported here is consistent with high levels of effectiveness reported in prior studies.

**Trial registration** ISRCTN95769543.

## Background

Around the world, people face structural barriers and legal restrictions that prevent access to high-quality abortion services. Even when abortion services are available in facility settings, some people prefer out-of-clinic abortion care for reasons related to privacy, autonomy, and concerns such as stigma, mistreatment, and high cost [1, 2]. The reasons that people attempt to self-manage abortion—defined here as ending one’s own pregnancy outside of a formal health-care setting—and the means by which they do so, vary widely by setting [1, 2]. The incidence of self-managed abortion is not well studied; estimates suggest that approximately 45% of abortions worldwide in 2010–2014 took place outside of a health facility [3]—and in some settings, the proportion may be closer to 70 or 80% [3, 4].

Given the barriers to abortion access in clinical settings, those in need of abortion care are increasingly obtaining mifepristone and misoprostol, World Health Organization (WHO) recommended medications for abortion [5], through informal sector routes including online services, pharmacies, hotlines, and drug sellers [6–10]. There is a growing body of literature from around the world indicating that when individuals have access to information about how to obtain the pills, how to take the pills, how to assess for completion, and warning signs that may indicate potential complications, the practice of self-managed medication abortion is safe and the experience satisfactory [8, 9, 11–15]. Much of the published literature has focused specifically on the experiences of self-managed medication abortion with telemedicine support from online websites that provide access to pills as well as information on how to use them via email communication; these studies report safe and effective abortion experiences [2, 8, 12, 13, 16–18].

Beyond online websites, people obtain information, medications, and support to self-manage their abortions in a variety of other ways. One emerging model is abortion accompaniment, where trained volunteers provide WHO-recommended evidence-based information about medication abortion, as well as physical and emotional support and person-centered care throughout the medication abortion process, over the phone or in person, outside of the formal health care system [15, 19–22]. This non-clinic based model of counselor-supported self-managed medication abortion care has come to be known as the “accompaniment model,” as people are virtually “accompanied” through the medication abortion process. Approximately fifty accompaniment groups are in operation around the world, providing support and information about self-managed medication abortion. However, despite the increasing number of abortion accompaniment groups worldwide, little research has documented the safety and effectiveness of the abortion accompaniment model. To our knowledge, only three studies have reported on outcomes of self-managed medication abortion among accompaniment group clients; all found high levels of abortion completion and few complications [15, 22, 23].

Of the data that do exist, however, there are important limitations, including a heavy reliance on retrospective records that were not collected for the purposes of research. Evidence suggests that a high proportion of abortions occur outside of the health care system and changing global dynamics may continue to shift more abortions outside of the healthcare system [24]. Well-designed, rigorously-collected data are needed to assess the safety and effectiveness of medication abortion administered completely outside of the formal healthcare system—such as the accompaniment model—to contribute to our understanding of de-medicalized models of abortion care [25].

To address this gap, we designed a pilot prospective observational study of the effectiveness and safety of self-managed medication abortion with accompaniment group support in three countries. We conducted the pilot study to inform the design and implementation of a larger, non-inferiority study to prospectively evaluate the effectiveness of self-managed medication abortion with accompaniment group support as compared to the effectiveness of medication abortion in a clinical setting. The primary aims of this pilot study were to (1) assess the feasibility of implementing a prospective study to recruit and follow callers to abortion accompaniment groups; and (2) evaluate hypotheses about the effectiveness and experiences of self-managed abortion under this model of care. While these data come from a pilot study, so do not represent results that are powered to make definitive statements about safety, these data provide foundational evidence for future studies on self-managed abortion, particularly as we find ourselves in a moment of history where delivery of healthcare services by the formal sector will require innovation, and where healthcare infrastructures around the globe will be challenged in unknowable ways [26, 27]. All of these factors could lead to an increase in incidence of and demand for self-managed abortion. These pilot study data represent some of the first prospective data on the effectiveness of self-managed abortion [2, 15, 22, 23], and provide insight into ways that healthcare systems could adapt to support those who choose to or need to self-manage abortions.

## Methods

### Study setting

A research consortium that includes researchers, advocates, and accompaniment providers collaboratively designed this study to ensure it reflected the priorities, experiences, and preferences of people who self-manage abortions with medication. Study investigators invited individual consortium members (included as co-authors) to participate based on their expertise in self-managed medication abortion and accompaniment models in a range of legal and cultural settings, to ensure the design of a study that reflected the lived experiences of people who self-manage, the accompaniers who support them, and contexts similar to those in which the study will take place.

Data for this study were collected in three countries located in South America, Southeast Asia, and West Africa. The names of the abortion accompaniment groups and their home countries are blinded based on the request of study implementation partners. The three included accompaniment groups were selected to represent variation in legal and sociocultural contexts with respect to abortion, and because they each had identified research as an important mechanism for informing policy and practice. The three accompaniment groups vary somewhat in their approach, but each involves an initial screening conversation with the pregnant person that takes place via secure messaging or a telephone call. During this screening conversation, the accompaniment counselor confirms that the person is seeking abortion for themselves, that the person is not being coerced, and that they have no known contraindications to medication abortion. Further, the counselor assesses the gestational age of the pregnancy based on either the date of last menstrual period as reported by the caller, or an independently acquired ultrasound. For callers who obtained an ultrasound, gestational age is based on the ultrasound dating. After confirming eligibility for medication abortion, the counselors then provide step-by-step instructions for how to use medication to induce abortion based on current WHO protocols (Appendix 1; of note, some of these regimens include off-label use of mifepristone and misoprostol), information on obtaining the medications, and highly detailed guidance on assessing abortion completion and potential warning signs of complications, as well as when formal healthcare may be needed. Accompaniment group staff are in frequent contact with callers during the medication abortion process to answer questions and provide support to the person self-managing an abortion. The accompaniment group in South America provides information primarily on a combined mifepristone and misoprostol regimen, while the groups in Southeast Asia and West Africa counsel on both a combined and misoprostol alone regimen, depending on which pills the caller is able to obtain.

### Study design and data collection

This pilot study was a prospective, observational study in which people who contacted an accompaniment group for information and support with self-managing a medication abortion were enrolled and followed for up to 6 weeks to assess their abortion outcome and experiences. As one of the primary aims of the pilot study was to assess feasibility, sample size was flexibly set to the number of people counselors could successfully recruit in 30 days. The study, like other medication abortion studies [28], was not powered to detect safety outcomes as major adverse events attributable to medication abortion are extremely rare [29]. Pilot study enrollment at each site was open for approximately 30 days in April and May of 2019. Participants were followed up to 6 weeks, with most followed for 3-weeks. We conducted the last follow-up interview in June 2019. Survey instruments were professionally translated into local languages as needed for each site, and were then pre-tested with four to five cognitive interviews in each country (13 total), and then updated accordingly.

During the initial counseling conversation, accompaniment counselors assessed all callers to the accompaniment group for eligibility for study participation during the 30 days. Eligibility criteria included: (1) having contacted the accompaniment group seeking information about induced abortion for their own pregnancy; (2) being at least 13 years of age; (3) being able to give informed consent; (4) being able to speak a local language; (5) meeting hotline eligibility criteria for starting the medication abortion process (i.e. no contraindications to medication abortion; Appendix 1); and (6) starting a new medication abortion process. Callers were excluded from the study if they (1) had taken medications in an attempt to end the current pregnancy within the 30 days prior to contacting the hotline; (2) were experiencing ongoing symptoms of spontaneous or induced abortion (bleeding, cramping) at the time of contacting the hotline; (3) had a known ectopic pregnancy; (4) did not want to share their contact information with study staff; (5) did not want to be contacted again by the hotline or by study staff; or (6) were not willing to comply with study procedures. Callers of any gestational age were eligible to participate in the study. Eligible participants were invited to participate by the accompaniment counselor at the end of the first counseling conversation. Participants who expressed interest proceeded through an informed consent conversation with detailed information about study participation, risks and benefits. All participants who gave their informed consent to participate were enrolled into the pilot study.

Immediately after enrollment, each participant answered baseline questions about their current pregnancy, reproductive history, contact information, and select sociodemographic characteristics. Follow-up surveys were completed by trained study coordinators at each site, recruited from trusted partner organizations, and employed full time on the research study for the duration of recruitment and data collection at each site. The first follow-up survey was conducted over the phone (voice-call or secure messaging) 1-week after the pills were scheduled to be taken. This 1-week follow-up inquired about obtaining the medications, medication type, detailed information on timing and route of administration, pain, bleeding and cramping during the abortion, and self-reported assessment of abortion completion. Two weeks after the first follow-up, approximately 3-weeks after the medication was taken, study coordinators contacted participants for a second-follow-up that included questions about any additional doses taken, warning signs of complications, completion of abortion, healthcare seeking, disclosure of the abortion, satisfaction with the accompaniment group, and emotions about the experience. Participants who reported they were no longer planning to take the pills, were asked why, and no further follow-up was conducted. Participants received an incentive in the form of phone credit for each survey completed. Study coordinators entered all survey data into Podio (https://podio.com/), a secure, online platform.

### Study measures

#### Effectiveness of self-managed medication abortion

The primary outcome of interest was effectiveness of self-managed medication abortion with accompaniment group support, defined as complete abortion at last study contact, without surgical intervention at any point. We classified an abortion process as “effective” if the participant responded “yes” to the question, “Do you feel that your abortion process is complete?” and did not report a surgical intervention when asked “At the health facility, what treatment did you receive?” (among participants who reported seeking medical care at a health facility during or after their abortion process). Participants also reported why they felt their abortion was complete, and if they had an ultrasound, or had taken a pregnancy test to confirm completion. We also calculated a secondary, more inclusive definition of “effective”—defined as a participant who was no longer pregnant at the end of follow-up, regardless of whether surgical intervention took place or not.

#### Complications

Warning signs of potential complications of self-managed medication abortion were assessed by asking participants to self-report any occurrence of (1) heavy bleeding that soaked more than two pads per hour for more than two hours, (2) pain that did not go away with the use of painkillers, (3) fever that lasted for over 24 h, and (4) foul smelling vaginal discharge. Participants were also asked about whether they experienced side effects at any point in the abortion process, including fever, diarrhea, nausea, vomiting, or dizziness, as well as signs of potential allergic reaction, including itchiness, difficulty breathing, sweaty hands, or face numbness.

Complications were identified based on participant self-report and receipt of treatment. Those who sought care at a facility at any point in the process were asked why they sought care and what treatment they received (surgical intervention, antibiotics, other medications, or observation).

### Analysis

We summarized baseline sociodemographic characteristics and data on reproductive history for the study population through measures of frequency and central tendency. We then calculated the proportion of participants who successfully obtained medications for abortion and used the medications to self-manage abortion at the 1-week follow-up, and described the medication abortion experience stratified by type of medication regimen. Finally, we calculated the proportion of participants who completed the abortion, the proportion who sought care, and the proportion that reported warning signs of complications by the 3-week follow-up. We conducted all analyses in Stata version 15.0. We double-entered study data for 46 participants (20%) to check for any systematic errors in data entry. We then conducted a sensitivity analysis to re-estimate the primary outcome under the conservative assumption that all those lost to follow-up had incomplete abortions.

## Ethical review

The overall study protocol was reviewed and approved by a central IRB of record based in the United States (the Allendale Investigational Review Board), by a local Institutional Review Board (IRB) as appropriate, and by a study-specific Data Monitoring and Oversight Committee (DMOC) with medication abortion experts from each study country, and a chair with research expertise in clinical medication abortion effectiveness studies. All participants provided verbal informed consent. Special emphasis was placed on potential participants less than 18 years of age, for whom counselors were trained to describe study participation in familiar terms, to ensure that any young person enrolled understood the risks and benefits, and was actively willing to participate.

## Results

### Sample characteristics

**Fig. 1:**
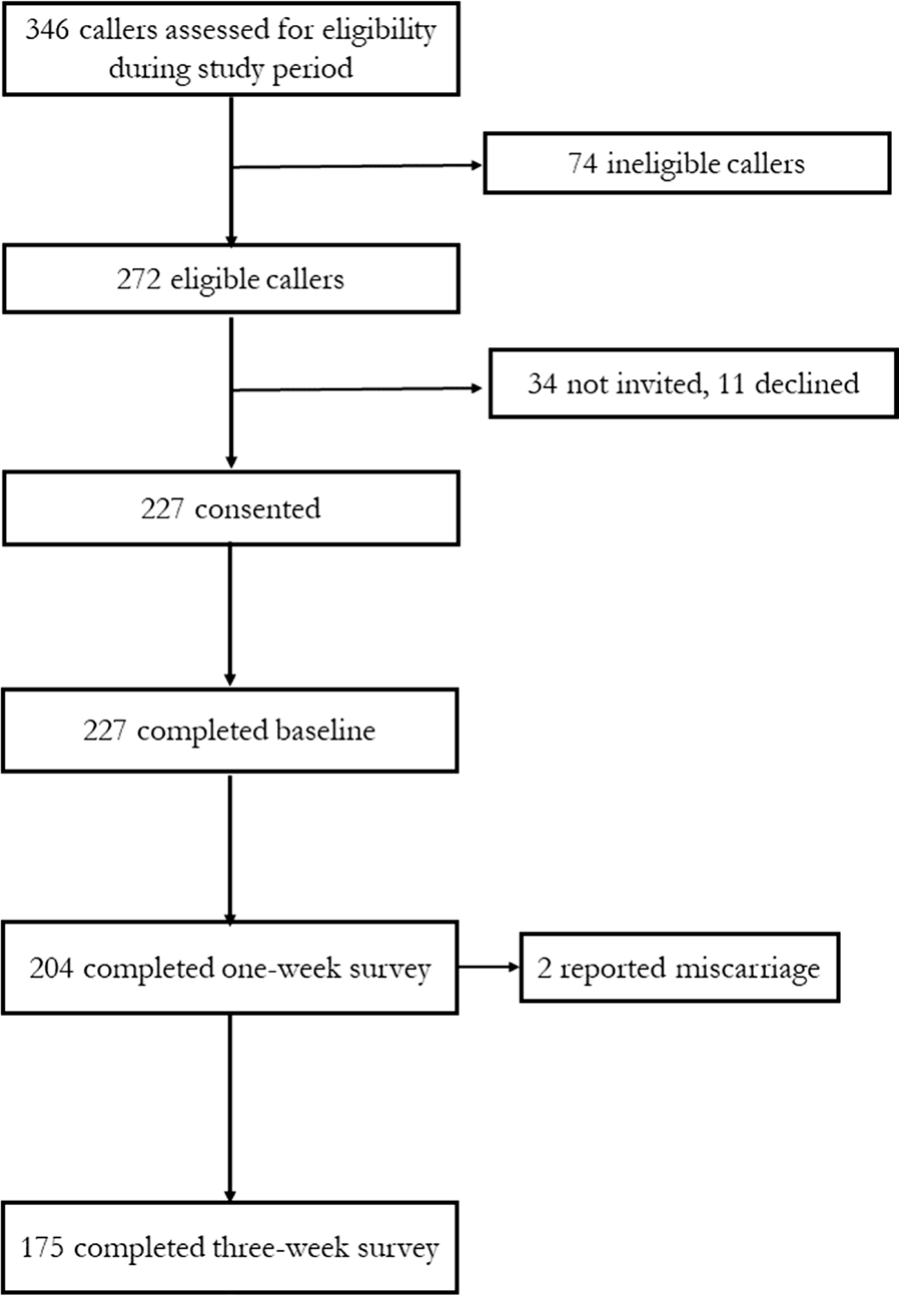
Screening and recruitment of callers to safe abortion accompaniment groups in South America, Southeast Asia, and West Africa over a one-month period

Study recruiters screened 346 callers for eligibility during the 30-day recruitment period (Fig. 1). Seventy-four callers (21%) were ineligible due to calling about something other than medication abortion, having already begun a medication abortion process, being outside of the accompaniment group gestational age range, being undecided about abortion, or unwilling to receive follow-up. During the initial counseling conversation prior to starting a medication abortion process, one participant described symptoms of a possible ectopic pregnancy. Upon hearing these symptoms, the counselor immediately referred the participant to a health facility for an ultrasound, where an ectopic pregnancy was confirmed, and the participant received appropriate and effective medical care. The study coordinator consequently classified this person as ineligible for study follow-up. Aside from this person, the study team enrolled 227 participants across the three sites. Two-hundred and four participants (90%) completed the 1-week survey, and 175 (77%) completed the 3-week survey.

At baseline, the majority of participants (75%) were less than 30 years of age, and 84% had at least some secondary education (Table 1). The majority of participants identified the pregnancy with a pregnancy test (89%), followed most closely by ultrasound (19%), and/or a late or missed menstrual period (15%). Only one participant (0.4%) reported a pregnancy that was not confirmed by a pregnancy test or ultrasound. At the time of enrollment, 68% of participants had a pregnancy of less than 8 weeks gestation, 21% between 8 and 9 weeks, 9% between 10 and 12 weeks, and 2% between 13 and 17 weeks. Only one study site measured method of gestational age ascertainment; most participants at this site assessed their gestational age based on date of the last menstrual period (79%). Ten percent of participants reported a prior attempt to end the current pregnancy, before contacting the accompaniment group. Prior attempts to end the pregnancy included ingesting herbs or using emergency contraception pills with intent to terminate.

**Table 1:** Sociodemographic characteristics of people pursuing self-managed medication abortion with accompaniment group support in three countries

	Total (n = 227)	
	n	%
Participant age (years)		
16–19	15	6.6
20–24	80	35.2
25–29	74	32.6
30–34	31	13.7
35–39	23	10.1
40–45	4	1.8
Level of education		
None	1	0.4
Primary	33	14.5
Secondary	70	30.8
> Secondary	120	52.9
Missing	3	1.3
Pregnancy characteristics	n	%
Ascertainment of pregnancy (select all)		
Pregnancy test	202	89.0
Ultrasound	44	19.4
Late/missed period	34	15.0
Pregnancy symptoms	28	12.3
Other	7	3.1
Gestational age		
< 8 weeks	154	67.8
8 or 9 weeks	47	20.7
10–12 weeks	20	8.8
13–17 weeks	4	1.8
Missing	2	0.9

### Use of medication abortion pills

At the 1-week follow-up, 203 participants (89% of those enrolled and 99% of those who participated in the 1-week survey) had obtained the medication—just under half of these 203 participants reported obtaining the medication from a pharmacy (n = 97, 49%). Two participants (1%) reported having had a miscarriage prior to taking the medications, and did not complete subsequent follow-ups; and 23 participants (10%) were lost to follow-up at 1 week. All but one participant who obtained the medications and completed the 1-week survey (n = 202) reported taking them by the 1-week follow-up (Table 2). Slightly over half of participants at the 1-week follow-up (n = 107, 53%) reported taking mifepristone and misoprostol in combination, 47% (n = 94) reported taking misoprostol alone, and one participant did not report medication used. Participants most commonly utilized sublingual administration for misoprostol (n = 339 of 370 doses, 92%).

**Table 2:** Medication abortion pills sourcing and utilization

Medication characteristics	One-week follow-up (n = 204)	
Have you gotten the pills yet?	n = 204	%
Yes	202	99.0
Yes, did not take b/c had a miscarriage	1	0.5
No, had a miscarriage prior to obtaining	1	0.5
How were the pills packaged?	n = 202	%
Blister pack	90	44.6
Loose pills	90	44.6
Missing	22	10.9
Have you taken the pills yet?	n = 202	%
Yes	202	100.0
Medication regimen	n = 202	
Mifepristone and misoprostol	107	53.0
Misoprostol alone	94	46.5
Missing	1	0.5
Misoprostol route of administration	n = 370	%
Sublingual	339	91.6
Buccal	15	4.1
Vaginal	12	3.2
Oral	2	0.5
Missing	2	0.5

Data reported by 204 participants approximately one-week after contacting an accompaniment group for support with self-managed medication abortion

### Completion of self-managed medication abortion

Three weeks after taking the medications, 95% of participants (n = 192) who took medications reported feeling that their abortion was complete at their last follow-up, and 94% (n = 189) felt their abortion was complete *and* had not reported surgical intervention (Table 3). Among the participants who had a complete abortion, three (1.5%) reported a surgical intervention to evacuate the uterus. Seven participants (3.5%) reported being “not sure” that their abortion was complete; four of these participants reported having felt the products of conception expel, but reported being unsure about the abortion completion because they had not yet taken a pregnancy test or had an ultrasound to confirm they were no longer pregnant. Another participant was unsure because she reported still feeling some “pregnancy symptoms,” and another was unsure because of a lack of confidence in medication abortion versus the certainty of surgical abortion. Three participants (1.5%) reported that they did not feel that their abortion was complete. One was still bleeding, and thus felt the process was not complete. The other two sought care in the formal healthcare system and were told by healthcare providers that their abortions were incomplete; however, healthcare providers did not intervene surgically or medically. Completion by gestational age is included in Appendix 2. We have no data on whether medications were taken or any subsequent outcomes for 23 participants (10%) who did not complete *any* post-enrollment follow-up. In a sensitivity analysis, we assumed that all of these lost participants (n = 23) obtained the medications and took them, but none had a complete abortion; under this assumption, the estimated proportion with a complete abortion at 3-weeks post enrollment would then fall to 85%, or 192 out of 225 participants (excluding the two who miscarried prior to taking the pills).

**Table 3:** Outcomes and experiences of self-managed medication abortion with accompaniment support

	All participants^a^ (n = 202)		Mife + Miso regimen (n = 107)		Miso only regimen (n = 94)	
Did you experience any bleeding?^b^	n	%	n	%	n	%
Yes	196	97.0	105	98.1	90	95.7
No	1	0.5	0	0.0	1	1.1
Missing	5	2.5	2	1.9	3	3.2
Did you experience any cramping/contractions?^b^						
Yes	192	95.0	99	92.5	92	97.9
No	7	3.5	7	6.5	0	0.0
Missing	3	1.5	1	0.9	2	2.1
Abortion outcome						
Complete abortion	192	95.1	103	96.3	88	93.6
Complete abortion and no surgical intervention	189	93.6	101	94.4	87	92.6
Unsure if abortion was complete^c^	7	3.5	2	1.9	5	5.3
Incomplete abortion^d^	3	1.5	2	1.9	1	1.1
If complete, how did you know? (select all)	n = 192		n = 103		n = 88	
Saw products of conception	91	47.4	57	55.3	34	38.6
Negative pregnancy test	36	18.8	9	8.7	27	30.7
Pregnancy symptoms ended	97	50.5	47	45.6	50	56.8
Ultrasound confirmed completion	33	17.2	10	9.7	23	26.1
Clinician told me	8	4.2	8	7.8	0	0.0
Accompanier/counselor told me	30	15.6	30	29.1	0	0.0

Data from 202 participants who took the medications**;** medication regimen is missing for one participant

^a^This column presents results from Mife + Miso users, Miso-only users, and one additional participant who did not specify medication regimen

^b^Reported at one-week follow-up

^c^The most common reason given for being “not sure” that an abortion was complete was because the participant had not yet taken a pregnancy test to confirm completion, despite having felt the products of conception expel

^d^One participant who said their abortion was not complete was still bleeding at the last follow-up

### Physical experience of self-managed medication abortion

Across both medication regimens, nearly all participants experienced bleeding and cramping during the abortion process (Table 3). The majority of participants reported using at least one technique to manage the pain: most commonly pain medications (n = 118 of 181, 65%), followed by methods of distraction, such as listening to music or watching television (n = 18, 10%) (Table 4). Participants also reported several side effects. Participants reported nausea most commonly (n = 111, 61%), followed by fever/chills (n = 110, 60%), diarrhea (n = 90, 50%), vomiting (n = 61, 34%), and dizziness (n = 7, 4%). Few participants reported signs of an allergic reaction to the medications.

**Table 4:** Physical experiences and health-care seeking during self-managed medication abortion

	Total	
	n	%
Side effects	n = 181	
Nausea	111	61.3
Any fever, for any duration	110	60.1
Diarrhea	90	49.7
Vomiting	61	33.7
Dizziness	7	3.9
Signs of potential allergic reaction	n = 181	
Itchiness	15	8.3
Difficulty breathing	2	1.1
Sweaty hands	1	0.6
Face numbness	1	0.6
Warning signs of complications	n = 181	
Foul smelling discharge	9	5.0
Bleeding 2 + hours	8	4.4
Pain that does not go away	7	4.1
Fever over 24 h	1	0.6
Pain management method	n = 181	
Pain medications	118	65.2
Distraction	18	9.9
Massage/hot water	10	5.5
Sought health care at a facility for any reason	n = 181	
Yes	60	33.1
No	114	63.0
Missing	7	3.9
Reason for seeking care	n = 60	
Confirm abortion	40	66.7
Symptoms/side effects (pain, fainting, bleeding)	5	8.3
Missing	15	25.0
Treatment received (select all)	n = 60	
Observation/confirmation of termination	50	83.3
Medications/tablets	5	8.3
Surgical intervention	3	5.0
Antibiotics	2	3.3
Disclosed to provider about abortion?	n = 60	
Yes	18	30.0
No	39	65.0
Missing	3	5.0

Data reported by participants at 1-week and 3-weeks following self-managed medication abortion with accompaniment support in Southeast Asia, South America and West Africa

### Safety of self-managed medication abortion

In the 3 weeks following medication abortion, some participants reported warning signs of potential complications (Table 4), most commonly foul smelling discharge (n = 9, 5%), bleeding that soaked more than two sanitary pads per hour for more than two hours (n = 8, 4%), pain that did not go away (n = 7, 4%), and fever that lasted more than 24 h (n = 1, 0.6%). Disproportionately more participants, however, reported seeking care during or after the self-managed medication abortion process (n = 60, 33%). Most of the participants that sought formal health care (n = 40 of 60, 67%) did so to confirm completion of the abortion, commonly at diagnostic laboratories rather than at a clinic or hospital. Of the participants who took medications, five (3%) received other medications, three (1.5%) received a surgical intervention (manual vacuum aspiration or dilation and curettage), and two (1%) received antibiotics. No major adverse events were reported, such as hysterectomy or death.

## Discussion

Findings from this pilot study indicate that recruitment and prospective follow-up of callers to abortion accompaniment groups is feasible, and provide preliminary evidence that self-managed medication abortion with accompaniment group support may be an effective model of abortion care. During approximately one month of follow-up, across three diverse settings, the majority of pilot study participants obtained medication abortion pills, took the medication abortion pills following information provided by accompaniment group counselors, and completed their abortion without the need for surgical intervention, and without major complications or other safety events.

The findings presented here build on a robust body of evidence that points to a similar conclusion: self-managed medication abortion with accurate information on how to use the pills can be effective and safe [2, 7, 8, 11, 14, 15, 22, 30–33]. This pilot study has demonstrated that research conducted in close partnership with groups that provide abortion accompaniment is a promising avenue to improve understanding of self-managed abortion. These groups facilitate the efficient identification of people self-managing their abortions (a group that has been historically difficult to identify and recruit for studies [25]) and enables deeper insight into these experiences given the naturally consistent interaction that often occurs between counselors and callers throughout the accompaniment process.

However, conducting rigorous evaluations of this de-medicalized model of care has a unique set of challenges, primarily the reliance of self-report for gestational age assessment and measurement of all primary outcomes. This approach reflects a high degree of confidence and trust in participant ability to self-assess study outcomes, as well as limitations imposed by legal restrictions in each country. Additionally, research from clinical settings has demonstrated the accuracy of report of last menstrual period as compared to ultrasound assessment [34–38]: the largest study to date found that only 3.3% of 4257 medication abortion clients from ten clinics across the United States had a gestational age beyond 63 days by ultrasound assessment but a gestational age below 63 days based on last menstrual period [34]. Even for those who may be off in their gestational age assessments, research has demonstrated that early medication abortion remains safe and highly acceptable without screening ultrasound [37]. Indeed, WHO technical guidance does not require ultrasound confirmation of gestational age for early medication abortion [5], and recent changes in the wake of the novel coronavirus 2019 pandemic have shifted clinical protocols in the United States, the United Kingdom, and elsewhere away from requiring ultrasounds or other confirmatory tests prior to medication abortion [39]. Furthermore, studies assessing the effectiveness of telemedicine for abortion, home administration of misoprostol, and telephone follow-up after a clinic-based medication abortion have demonstrated the reliability of self-report of completion based on structured criteria [40, 41].

Our study also suffered from loss-to-follow-up. To understand the extent to which this could have biased results, we conducted a sensitivity analysis in which we made the most conservative assumption that all participants who were lost-to-follow-up had a failed abortion. Under this conservative assumption, the overall effectiveness of self-managed abortion with accompaniment support is 85%—which is similar to the efficacy of misoprostol alone demonstrated in clinical trials [42]. Additionally, the proportion lost to follow-up is comparable to typical loss to follow-up among medication abortion clients in clinic visits in the United States [43]. However, given the restrictive legal and social environments in which the study took place, the accompaniment groups report that individuals who are lost to follow-up tend to be those with successful abortions who need no additional support, while those in need of information on accessing formal health care, or who experience a failed abortion, are more likely to stay in contact as they are often the only source of care or information for these people. Accompaniment groups report very few instances of complications being reported more than one month after a person takes the pills. Additional limitations include the different medication regimens and screening protocols used across sites, as well as the range of gestational ages. These limitations, however, are balanced by several key strengths, including the study design which allows prospective assessment of abortion experiences with a high degree of detail, as well as the range of settings and contexts covered by participants from the three countries. The generalizability of study results to other self-managed medication abortion settings may depend on the quality of information and counseling provided, and the medication protocols recommended.

## Conclusions

Findings from this prospective, observational, multi-country pilot study demonstrate that prospective data collection among people who self-manage abortions in restrictive contexts is feasible. Further, findings are consistent with the hypothesis that self-managed medication abortion with accompaniment group support is safe and effective. These data will inform a larger, prospective, non-inferiority study to strengthen the findings presented here. These results offer a contribution to policy makers and professional bodies as they imminently consider revisions to task-shifting and other medication abortion provision guidelines, as well as if and how to support the de-medicalization of medication abortion services as healthcare infrastructures around the globe adapt in the wake of a global pandemic.

## Data Availability

The datasets generated during the current study are not publicly available due to concerns regarding confidentiality but are available from the corresponding author on reasonable request.

## Appendix 1: Medication abortion protocols as of March 2019 and contraindications

### South America

Mifepristone + misoprostol for pregnancies up to 84 days: oral/sublingual

- Swallow 1 tablet of mifepristone (200 mg) with a glass of water.
- After 36–48 h, put 4 pills of misoprostol (800mcg) under the tongue (sublingual) and let them dissolve for 30 min, keep swallowing saliva until the pills dissolve.

Mifepristone + misoprostol for pregnancies up to 84 days: oral/vaginal

- Swallow 1 tablet of mifepristone (200 mg) with a glass of water.
- After 36–48 h, put 4 pills of misoprostol (800mcg) inside the vagina, fairly deep into the vagina. Rest for one hour with your legs up. (Before placing the pills, you can splash them with water).

#### Contraindications

If a caller reports any of an: c-section within the past six months, ectopic pregnancy, chronic adrenal failure, long term corticosteroid therapy, intolerable known allergy to mifepristone or misoprostol, current use of intrauterine device (IUD), or hemorrhagic disorders, or has not chosen to terminate the pregnancy of their own free will, the caller is deemed ineligible for medication abortion.

### Southeast Asia

Mifepristone + misoprostol for pregnancies up to 84 days

- Swallow 1 tablet of mifepristone (200 mg) with a glass of water
- After 24 h, put 4 pills of misoprostol (800mcg) under the tongue (sublingual) and let them dissolve for 30 min, keep swallowing saliva until the pills dissolve.
- *If no signs of reaction, side effects and expulsion has not occurred after 3 h**, *put 2 pills of misoprostol (400mcg) under the tongue (sublingual) and let them dissolve for 30 min, keep swallowing saliva until the pills dissolve*.*
- Take 2 pills misoprostol the same way every 3 h until the products of conception expel.

Misoprostol alone for pregnancies up to 84 days

- Put 4 pills of misoprostol (800mcg) under the tongue (sublingual) and let them dissolve for 30 min, keep swallowing saliva until the pills dissolve. Wait for 3 h.
- If no signs of reaction, side effects and expulsion has not occurred, after 3 h, put add another 2 pills (400 mcg) the same way.
- Repeat 2 pills the same way every 3 h until when the product of conception expel.

Mifepristone + misoprostol for pregnancies beyond 84 days

- Swallow 1 tablet of mifepristone (200 mg) with a glass of water.
- After 24 h, Put 2 pills of misoprostol (400mcg) under the tongue (sublingual) and let them dissolve for 30 min, keep swallowing saliva until the pills dissolve. Wait for 3 h.
- Repeat 2 pills the same way every 3 h and *stop* when the product of conception expelled.

Misoprostol only for pregnancies beyond 84 days

- Put 2 pills of misoprostol (400mcg) under the tongue (sublingual) and let them dissolve for 30 min, keep swallowing saliva until the pills dissolve. Wait for 3 h.
- Repeat 2 pills the same way every 3 h and *stop* when the product of conception expelled.

#### Contraindications

If a caller reports any of the following: c-section within the past six months, ectopic pregnancy, chronic adrenal failure, long term corticosteroid therapy, intolerable known allergy to mifepristone or misoprostol, current use of IUD, or hemorrhagic disorders, the caller is deemed ineligible for medication abortion, but may still proceed with counselor and referral for surgical abortion services. Anyone who is not interested in terminating of their own free will is ineligible.

### West Africa

The hotline in West Africa provides information to callers based on the WHO protocol for medication abortion:

*Misoprostol only for pregnancies up to 84 days*

12 misoprostol pills of 200 mcg each (2400 mcg of misoprostol total)

- Put 4 pills (800mcg) under the tongue (sublingual) and let them dissolve for 30 min, keep swallowing saliva until the pills dissolve. Wait for 3 h.
- After 3 h, put the second dose of 4 pills (800 mcg) under the tongue and let them dissolve for 30 min, keep swallowing saliva until the pills dissolve. Wait for 3 h.
- After 3 h, put a third dose of 4 pills (800 mcg) under the tongue and let them dissolve for 30 min, keep swallowing saliva until the pills dissolve.

*Mifepristone + misoprostol for pregnancies up to 84 days*

1 mifepristone tablet of 200 mg and 4 misoprostol tablets of 200 mcg each (800 mcg of misoprostol total)

- Swallow 1 tablet of mifepristone (200 mg) with a glass of water
- After 24 h, put 4 pills (800 mcg) of misoprostol between the gum and the cheek (buccal), two on the left side and two on the right side.
- All four pills should be left in the mouth for approximately 30 min to dissolve.

#### Contraindications

If a caller reports any of: a current IUD, allergy to misoprostol, current sexually transmitted infection, an ectopic pregnancy, or has not chosen to terminate the pregnancy of their own free will, the caller is deemed ineligible for medication abortion.

## Appendix 2. Self-managed medication abortion outcomes by gestational age

**Table:**

	< 8 weeks (n = 141)		8–9 weeks (n = 40)		10–12 weeks (n = 16)		13–17 weeks (n = 3)		GA missing (n = 2)	
	n	%	n	%	n	%	n	%	n	%
Abortion outcome										
Complete abortion	134	95.0	37	92.5	16	100.0	3	100.0	2	100.0
Complete abortion, no surgical intervention	133	94.3	36	90.0	15	93.8	3	100.0	2	100.0
Unsure if abortion was complete	5	3.5	2	5.0	0	0.0	0	0.0	0	0.0
Incomplete abortion	2	1.4	1	2.5	0	0.0	0	0.0	0	0.0
Reported a miscarriage prior to taking pills	2		0		0		0		0	
Lost to follow-up prior to taking pills	11		7		4		1		0	

## Abbreviations

DMOC: Data monitoring and oversight committee
WHO: World Health Organization
IRB: Institutional Review Board
